# PMSF Attenuates Morphine Antinociceptive Tolerance and Dependence in Mice: Its Association with the Oxidative Stress Suppression

**DOI:** 10.22037/ijpr.2020.112936.14038

**Published:** 2021

**Authors:** Ehsan Asadi Akbarabadi, Hossein Rajabi Vardanjani, Shahrzad Molavinia, Marzieh Pashmforoosh, Mohammad Javad Khodayar

**Affiliations:** a *Student Research Committee, Ahvaz Jundishapur University of Medical Sciences, Ahvaz, Iran. *; b *Medical Plants Research Center, Basic Health Sciences Institute, Shahrekord University of Medical Sciences, Shahrekord, Iran. *; c *Toxicology Research Center, Medical Basic Sciences Research Institute, Ahvaz Jundishapur University of Medical Sciences, Ahvaz, Iran. *; d *Department of Toxicology, School of Pharmacy, Ahvaz Jundishapur University of Medical Sciences, Ahvaz, Iran. *; e *Behbahan Faculty of Medical Sciences, Behbahan, Iran.*

**Keywords:** Morphine, Tolerance, Dependence, Oxidative stress, PMSF, Mice

## Abstract

Opioids use has been limited due to tolerance and dependence as major unwanted effects. Previous evidence has shown that targeting endocannabinoid signaling can prevent the development of opioid tolerance and dependence. This study was designed to evaluate the effect of phenylmethylsulfonyl fluoride (PMSF), an inhibitor of fatty acid amide hydrolase (FAAH), on morphine antinociceptive tolerance and physical dependence in mice. The antinociceptive effects of PMSF at the doses 60, 120, and 300 mg/kg were investigated. Results showed that PMSF has a notable antinociceptive effect at doses 120 and 300 mg/kg. The dose of (60 mg/kg, i.p.) PMSF was considered as a sub-antinociceptive dose. Morphine tolerance and dependence were induced by twice-daily injection of morphine (10 mg/kg, s.c.) for 10 consecutive days and the last dose on day 11. Tolerance was assessed by the hot-plate test and dependence by naloxone-precipitated morphine withdrawal signs. In the brain, oxidative stress markers include activities of glutathione peroxidase, catalase, superoxide dismutase, and levels of malondialdehyde and glutathione were determined. A sub-antinociceptive dose (60 mg/kg) of PMSF could reduce tolerance in both acute and chronic methods of administration. However, alleviation of dependence and suppression of oxidative stress markers occurred in the chronic administration of PMSF. In conclusion, it seems that PMSF can suppress morphine tolerance and dependence. However, more studies are needed to clarify its mechanism.

## Introduction

Drug addiction is a disorder characterized by changes in the central nervous system. It can be accompanied by intracellular alteration of specific brain neurons after frequent exposure to these drugs (1). Repeated exposure to opioids is limited because of tolerance and risk of dependence (2). It has been demonstrated that the compulsive desire for drug use such as morphine occurs due to the neuroadaptation during drug addiction processes which will afford the need for increasing doses to get favorable effects (3). The most common adverse effects occur after morphine dependence, including anxiety, restlessness, dilated pupils, excessive sweating, respiratory depression, hypertension, muscle pain, and digestive problems (4, 5). The development of morphine tolerance and dependence is associated with receptor-mediated neuronal activity caused by chronic exposure. The symptoms of tolerance are assessed by the classical method of evaluating antinociceptive activities. At the same time, signs of dependence are examined by assessing withdrawal after administration of the narcotic antagonist (6, 7). The mechanisms of tolerance and dependence involve one or more mechanisms, including desensitization and down-regulation of opioid receptors, uncoupling of opioid receptors-G protein, and up-regulation of the cAMP signaling (8, 9). Morphine can cause oxidative stress under certain conditions by increasing the formation of different kinds of free radicals, including superoxide radical anion and nitric oxide (NO) (10). Based on the previous reports, NO plays an important function in the development of tolerance and dependence of morphine via increasing the concentration of cGMP by activating guanylyl cyclase. (11–13). Some reports have indicated that the free radical scavenging agents could be potent ways to inhibit morphine tolerance and withdrawal syndrome (14). Fatty acid amide hydrolase (FAAH), which has been previously termed anandamide amidase, is an integral membrane enzyme that inactivates anandamide (partial CB1 agonist) (15). As a result, inhibition of FAAH increases anandamide levels and consequently potentiates endocannabinoid signaling (16, 17). It has been demonstrated that the enzymatic activity of FAAH is sensitive to inhibition by the serine protease inhibitor phenylmethylsulfonyl fluoride (PMSF) (18, 19). PMSF is an inhibitor of the enzyme that inhibits some thiol proteases, non-protease enzymes, and acetylcholinesterase. PMSF reacts with serine residues that cause irreversible inhibition of the enzyme (20–22). This compound can enhance the level of anandamide by preventing its metabolism (23). Some behavioral and antinociceptive studies in animal models have been shown that FAAH inhibitors reduce symptoms of opioid withdrawal and enhance the antinociceptive properties of morphine (24, 25). These findings support the efficacy of inhibition of FAAH for the prevention of dependence and tolerance of drugs. Based on the above findings, we propose that FAAH as a therapeutic target on the morphine-related effects. Thus, in the present study, we examined the effect of FAAH inhibitor, PMSF on morphine antinociceptive tolerance and dependence in mice.

## Experimental

*Animals*

Adult male mice (20–22 g) were obtained from the central animal house of Jundishapur University of Medical Sciences (Ahvaz, Iran). They were placed at 23 ± 2 °C and 12 h light/dark cycles (light from 7:00 to 19:00) with free access to water and food. Animals were randomly divided into groups of 8, acclimatized to the laboratory environment for at least one week before the experiments. The animals were used only once throughout the experiments. Animal care and experimental procedures were carried out in accordance with the standards of the Ethical Committee of Animal Experimentation of Ahvaz Jundishapur University of Medical Sciences (IR.AJUMS.REC.1395.38). All behavioral tests were performed by a blinded investigator.

*Drugs*

Morphine sulfate (Darupakhsh Co, Tehran, Iran) and naloxone hydrochloride (Tolidaru Co, Tehran, Iran) were dissolved in physiological saline (0.9% NaCl). PMSF (Sigma, USA) was suspended in a physiological saline solution containing sesame oil. Morphine was administered subcutaneously (s.c.), and PMSF was administered intraperitoneally (i.p.).

*The hot-plate test*

This assay measures analgesic activity according to the study of Eddy *et al*. (26). All animals were placed on a 55 ± 1 °C hot plate, and the latency time to either jumping or licking was recorded. The cut-off time was set as 15 s to protect mice from any damage. The antinociceptive effects of a single dose of morphine (10 mg/kg s.c.) and PMSF (60, 120, and 300 mg/kg) were determined.

*Induction of morphine tolerance and dependence and evaluation*

Tolerance and dependence on morphine were induced by morphine 10 mg/kg s.c., twice daily for 10 days and the last dose on day 11, with modifications based on the study of Joshi *et al*. (7). Tolerance to morphine was evaluated by hot-plate antinociceptive test and physical dependence through naloxone (4 mg/kg)-precipitated morphine withdrawal signs. Withdrawal signs include jumping and weight loss were evaluated.

*Experimental design*

**Figure 1:**
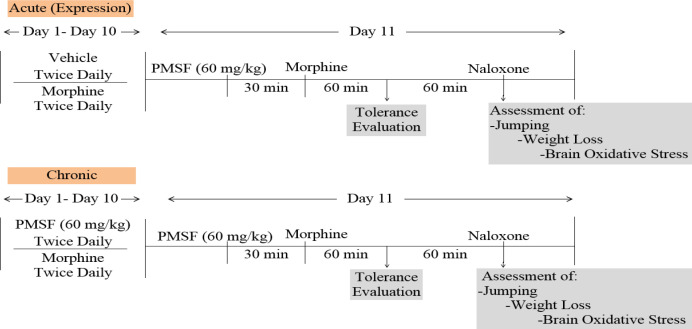
Timeline treatment with PMSF on acute and chronic morphine tolerance and dependence in morphine tolerant and dependent mice

**Figure 2:**
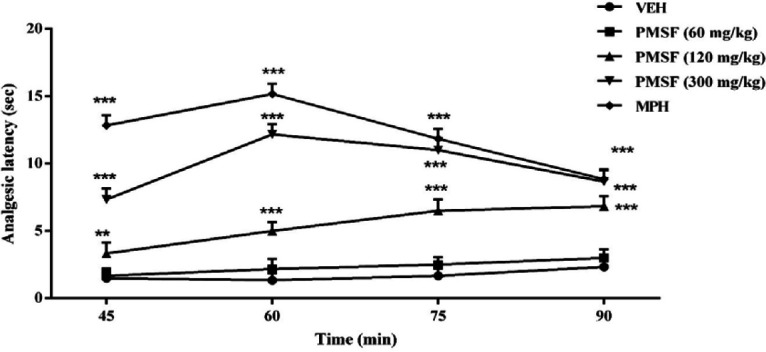
Effects of PMSF and morphine (MPH) on nociceptive responses in the mouse hot-plate test. Vehicle (VEH), PMSF, and MPH were administered 30 min before beginning the test. The latencies of nociceptive responses were measured 45, 60, 75, and 90 min after PMSF administration

Experiments were made to evaluate the effects of PMSF on nociception and its role in morphine analgesia. Then, the sub-effective dose of PMSF has been studied on the acute and chronic morphine tolerance and dependence in mice and the associated oxidative stress. Two schedules of studies were conducted to examine PMSF effects on acute and chronic morphine tolerance and dependence (Figure 1). In this way, experiments were carried out as follows: 1) Assessment of antinociceptive effects of PMSF at the doses of 60, 120, and 300 mg/kg in comparison of morphine in the hot-plate test at the times of 45, 60, 75, and 90 min after PMSF or morphine (Figure 2).

**Figure 3:**
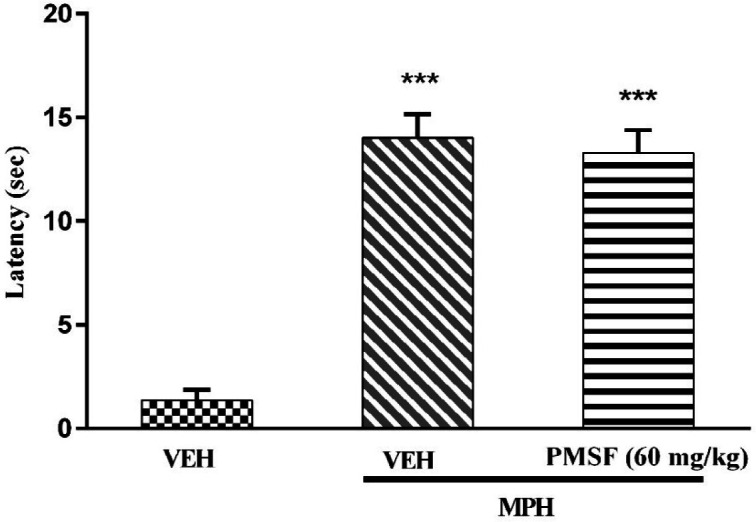
PMSF effect on the analgesic latency of acute morphine. The latency of nociceptive responses was measured 60 min after MPH administration. To assess the effects of PMSF on acute morphine-induced antinociception in the hot-plate test, the animals received a dose of PMSF (60 mg/kg, i.p.), 30 min before morphine (10 mg/kg, s.c.). Data are expressed as the means ± SEM. Each group consisted of 8 mice. ^***^P < 0.001 compared to vehicle control

**Figure 4:**
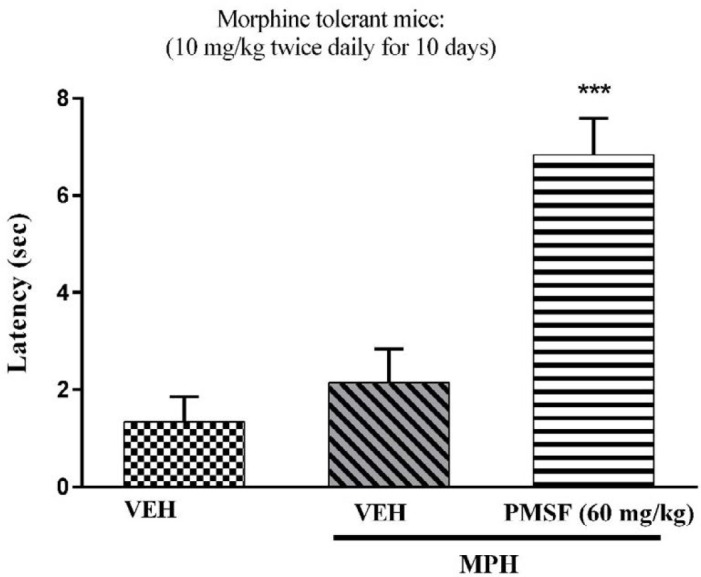
Effect of acute administration of PMSF on the expression of morphine tolerance in mice. To evaluate whether PMSF could reverse morphine tolerance in mice, the animal received morphine (10 mg/kg, s.c.) twice daily for 10 days and on the test day (day11), PMSF (60 mg/kg, i.p.) was administrated 30 min before morphine injection. The hot-plate test was carried out to evaluate the effect of PMSF on the expression of morphine tolerance. Each point represents the means ± SEM. (n = 8). ^***^P < 0.001 *vs.* vehicle-treated group (ANOVA Tukey's multiple comparison test)

**Figure 5:**
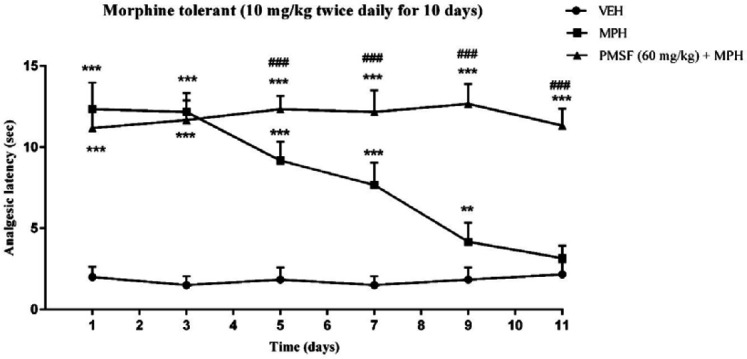
Effect of chronic administration of PMSF on the development of tolerance to the analgesic effect of morphine in mice. To evaluate whether PMSF could reverse morphine tolerance in mice, PMSF (60 mg/kg, i.p.) was administered 30 min before morphine (10 mg/kg, s.c.) twice daily for 11 days. The analgesic effect was recorded on the 1^st^, 3^rd^, 5^th^, 7^th^, 9^th,^ and 11^th^ days, 60 min after morphine injection in the hot-plate test. Each point represents the means ± SEM. (n = 8). ^**^P < 0.01, ^***^P < 0.001 *vs.* saline-treated group; ^###^*P < *0.001 *vs.* morphine alone treated group (Bonferroni's multiple comparison test)

**Figure 6:**
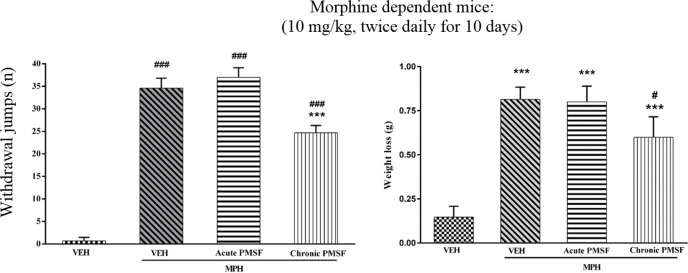
Effect of acute or chronic administration of PMSF on naloxone-induced withdrawal jump and weight loss in morphine-dependent mice. In the chronic study, animals received PMSF (60 mg/kg, i.p.) twice daily for 11 days 30 min before each morphine (10 mg/kg, s.c.) and in the expression phase, animals received PMSF (60 mg/kg, i.p.) on the 11^th^ day, 30 min before last morphine (10 mg/kg, s.c.) injection. Naloxone (4 mg/kg) was injected into mice on the 11^th^ day, 2h after morphine. Each point represents the means ± SEM. (n = 8). ^***^P < 0.001 *vs.* morphine treated group alone,^ ###^*P < *0.001 *vs.* vehicle-treated group (ANOVA Tukey's multiple comparisons test)

**Figure 7:**
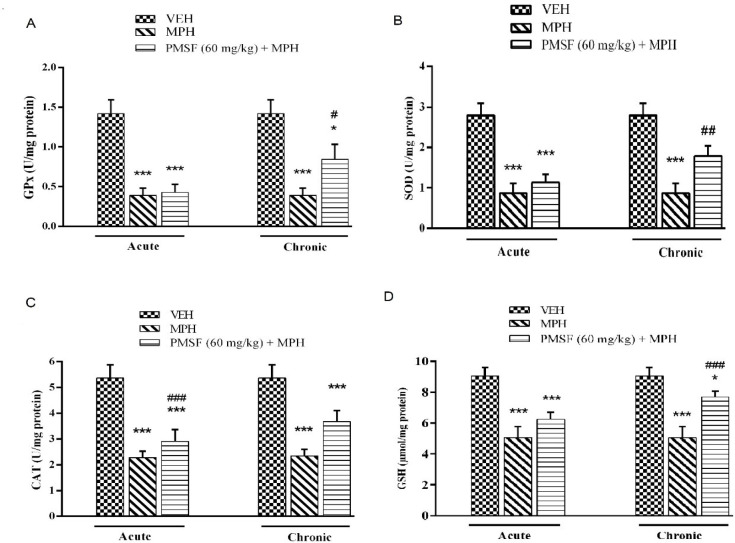
Effect of the chronic or acute administration of PMSF on naloxone-induced alterations in the brain GPx (A), SOD (B), CAT (C), and GSH (D) levels in morphine-dependent mice. In the chronic study, animals received PMSF (60 mg/kg, i.p. twice daily for 10 days) 30 min before morphine (10 mg/kg, s.c. twice daily for 10 days) and on the 11^th^ in the expression phase, animals received PMSF (60 mg/kg, i.p.) 30 min before last morphine (10 mg/kg, s.c.) injection. In the expression phase, animals received morphine (10 mg/kg, s.c. twice daily for 10 days) and on the 11^th^ day PMSF (60 mg/kg, i.p.) 30 min before last morphine (10 mg/kg, s.c.) injection. Naloxone (4 mg/kg) was injected into mice on the 11^th^ day, 2h after morphine

**Figure 8:**
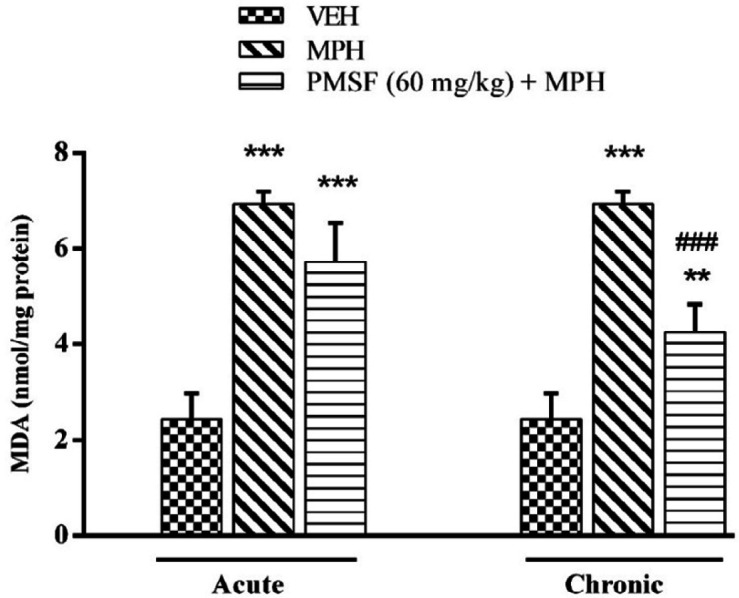
Effect of chronic or acute administration of PMSF on naloxone-induced alterations in the brain MDA level in morphine-dependent mice. In the chronic study, animals received PMSF (60 mg/kg, i.p. twice daily for 10 days) 30 min before morphine (10 mg/kg, s.c. twice daily for 10 days) and on day 11 in the expression phase, animals received PMSF (60 mg/kg, i.p.) 30 min before last morphine (10 mg/kg, s.c.) injection. In the expression phase, animals received morphine (10 mg/kg, s.c. twice daily for 10 days) and on the 11^th^ day PMSF (60 mg/kg, i.p.) 30 min before last morphine (10 mg/kg, s.c.) injection. Naloxone (4 mg/kg) was injected into mice on the 11^th^ day, 2 h after morphine. Each point represents the means ± SEM. (n = 8). ^*^P < 0.05, ^**^P < 0.01, ^***^P < 0.001 *vs*. vehicle-treated group, ^###^*P < *0.001 *vs*. morphine-treated group, alone. (ANOVA Tukey's multiple comparisons test)

2) Evaluation of sub-effective dose of PMSF (60 mg/kg) on morphine antinociception in the hot-plate test. Morphine analgesia was recorded 30 min after PMSF (Figure 3). 3) The effects of PMSF on acute and chronic morphine tolerance were evaluated. Tolerance was evaluated 60 min after morphine injection (Figures 4 and 5). 4) The effects of PMSF on acute and chronic morphine dependence were assessed. Naloxone-precipitated morphine withdrawal signs include the number of jumps and weight loss were recorded and then the brain oxidative stress merkers were assessed (Figures 6-8).

*Measurement of oxidative stress factors*

After the behavioral testing, the animals were sacrificed by cervical dislocation. Then, the whole brain was removed, homogenized and then centrifuged at 4,000 rpm for 15 min at 4 °C to measure oxidative stress parameters. Activities of antioxidant enzymes consisting of superoxide dismutase (SOD), catalase (CAT) and glutathione peroxidase (GPx) and the levels of malondialdehyde (MDA) and glutathione (GSH) were measured using the kit.

*Statistical analysis*

Data are shown as means ± SEM. Variables compared using a one-way analysis of variance followed by Tukey’s post hoc test and two-way ANOVA with Bonferroni’s multiple comparison test. Statistical significance was set at *P *< 0.05.

## Results

*Effects of PMSF on nociceptive responses*

The results suggest that the administration of the PMSF doses (120 and 300 mg/kg, i.p.) led to significant changes in the analgesic latency time (*P < *0.01 and *P < *0.001), while administration with PMSF (60 mg/kg i.p.) had no significant difference (Figure 2). Thus, PMSF at the dose of 60 mg/kg was considered as the sub-effective dose.

*Effect of PMSF on acute nociceptive behavior of morphine*

Results showed that PMSF (60 mg/kg) did not change the latency of nociceptive response in the hot-plate test. As shown in Figure 3, co-administration of PMSF with morphine could not induce any significant effect in comparison to the group receiving morphine alone.

*Effect of PMSF on the acute morphine tolerance*

As shown in Figure 4, co-administration of PMSF with morphine significantly increased analgesic effect of morphine during the development of morphine tolerance compared with the group receiving morphine alone (*P < *0.001). The results revealed that PMSF was able to reverse the expression of morphine tolerance in mice.

*Effect of PMSF on chronic morphine tolerance*

As depicted in Figure 5, the injection of morphine for 11 days caused an attenuation in analgesic effect compared to normal mice (saline group) (*P < *0.01 and *P < *0.001). While chronic co-administration of PMSF with morphine significantly diminished the chronic antinociceptive tolerance with comparison to morphine-treated animals (*P < *0.001).

*Effect of PMSF on naloxone-induced withdrawal jumps and weight loss in morphine-dependent mice*

Results show that jumping behavior and weight loss were not affected in the expression phase, suggesting that PMSF did not have possible effects on the expression of naloxone-precipitated withdrawal signs. As shown in Figure 6, administration of naloxone induced jumping and weight loss in dependent mice on the 11th day as compared to control animals (*P < *0.001). In the chronic study, administration of naloxone significantly inhibited jumping and weight loss in comparison to the morphine group (*P < *0.001).

*Effect of chronic or acute administration of PMSF on naloxone-induced alterations on GPx, SOD, CAT, and GSH levels*

As illustrated in Figure 7, the result revealed that the level of antioxidant factors including GPx, SOD, CAT, and GSH decreased in the brain of morphine-dependent mice after naloxone administration as compared with the vehicle-treated control group (*P < *0.001). In contrast, chronic administration of PMSF caused an increase in the level of GPx, SOD, CAT, and GSH. These effects compared to the morphine group (*P < *0.05, *P < *0.01, and *P < *0.001, respectively) and showed significant enhancement. In contrast, acute administration PMSF had no effects.

*Effect of chronic or acute administration of PMSF on naloxone-induced alterations* *on MDA level*

As illustrated in Figure 8, a significant increase in the level of the brain MDA was seen in the morphine-treated animals compared to that in vehicle-treated mice (*P < *0.001). On the other hand, lipid peroxidation was significantly declined by chronic co-administration of PMSF and morphine in comparison to morphine-treated animals (*P < *0.001). In contrast, the expression phase showed no significant difference compared to the morphine group.

## Discussion

More recent evidence has indicated that opioid receptors have the main role in the peripheral and central neurons of pain (27). Opiate drugs, such as morphine, are the most effective therapy for severe pain, but these compounds are remarkably affected by the development of analgesic tolerance, physical and psychological dependence (28). In the current study, the effects of PMSF on nociceptive responses in the hot-plate test were examined and the antinociceptive threshold was measured in animals. The results demonstrate that PMSF caused antinociception at the doses 120 and 300 mg/kg, whereas the dose of 60 mg/kg PMSF did not change the antinociceptive threshold. Accordingly, the dose of 60 mg/kg was considered as sub-effective dose (Figure 2). It has been shown that parenteral administration of PMSF can cross the blood-brain barrier and is transported into the brain (29). In an investigation, it has been demonstrated the influence of PMSF on anandamide brain levels and pharmacological effects. The result of this survey showed that pretreatment with PMSF was able to induce antinociception by enhancing brain levels of anandamide (17). Antinociceptive effects of PMSF may be mediated through an increase in the level of endocannabinoid, especially anandamide. Anandamide as the agonist of cannabinoid CB1 and CB2 receptors induces analgesia (30). Moreover, PMSF can inhibit serine proteinases, which would normally separate endorphins into inactive pieces (31). The function of endorphins is similar to opioids such as morphine. Endorphins delay perception of pain through interaction with the group of opioid receptors and neurons in the brain that modulate the gamma-aminobutyric acid (GABA) transmission (32, 33). The tolerance and dependence capacity of endorphins is a documentary reality and the mechanism of the expansion of tolerance to endorphins is likely to be relevant to the underlying tolerance to opiates (34). To evaluate the morphine tolerance in the pain model, the selected dose of PMSF should not cause an analgesic response, and also does not change the analgesic effect of morphine. For this purpose, the effects of PMSF on the acute nociceptive behavior of morphine were assessed in mice. The results indicated that the sub-effective dose of PMSF does not affect the antinociceptive behavior of morphine (Figure 3). In this paper, the effect of acute administration of PMSF on the expression of morphine tolerance on day 11 shows that PMSF reverse tolerance to morphine (Figure 4) and chronic use of PMSF resolved morphine tolerance at the days 5, 7, 9 and 11 (Figure 5). Alleviation of acute and chronic morphine tolerance by PMSF may be related to the endocannabinoid Interactions with the opioids system. In this regard, it was shown that the correlation between receptor affinity and potency of anandamide suggests that is probably enhanced with the administration of PMSF (35). In another study, Compton* et al *indicated that after the administration of PMSF, increased the activity of anandamide in an analgesic test (23). The naloxone jump and weight loss test are as a measure of recognition of opiate dependence or degree of severity of the withdrawal syndrome (36). It was demonstrated that the effect of naloxone, a potent opiate receptor blocker, could be attributed to the removal of the effects of opioid peptides (37). After naloxone administration, significant behavioral alterations such as jumping and weight loss were observed in morphine administered mice, confirming the development of morphine dependence. Chronic administration of PMSF decreased naloxone-induced withdrawal jump and weight loss in morphine-dependent mice. In contrast, acute administration PMSF had no effects (Figure 6). Previous studies have been shown that morphine administration produces ROS. Besides it also able to increases oxidative harm to the DNA, protein, and lipid. Morphine decreases in the activities of the antioxidant enzymes include GPx, SOD, CAT, and also GSH content (38–40). The findings showed that treatment of morphine-dependent mice with naloxone causes an increase in the level of MDA, and a reduction in endogenous antioxidants such as GPx, SOD, CAT, and GSH level of brain tissue. Chronic administration of PMSF induced marked effects possibly through alterations in antioxidant status (including decreasing MDA value, as well as increase in activities of GPx, SOD, CAT enzymes and GSH levels) against the effects of morphine in the brain. Acute administration of PMSF, unlike chronic, did not significantly change oxidative stress markers in morphine-dependent mice (Figure 7 and Figure 8). In the present study, the effect of PMSF as a FAAH inhibitor was evaluated on morphine-induced tolerance and dependence in mice. Inhibitors of FAAH can prohibit the breakdown of endogenous ligands for cannabinoid receptors. The chronic increase of anandamide induced by the FAAH inhibitors diminishes the development of morphine tolerance and dependence than the chronic use of cannabinoid-receptor agonists (41). A growing body of evidence reveals that the targeting of FAAH can be a strategy for the reduction in inflammation, pain, and neuropsychiatric disorders (42). Given this information, it can be confirmed that PMSF can improve the unreliable effects of opioids.

Data are expressed as the means ± SEM. Each group consisted of 8 mice. ^**^P < 0.01, ^***^P < 0.001 compared to the vehicle control (two-way ANOVA Tukey's multiple comparison test).

Each point represents the means ± SEM. (n = 8).^*^P < 0.05, ^***^P < 0.001 *vs.* vehicle-treated group, ^#^*P < *0.05, ^##^*P < *0.01, ^###^*P < *0.001 *vs.* morphine treated group, alone. (ANOVA Tukey's multiple comparison test)

## Conclusion

In summary, PMSF can be recommended for co-treatment with opioids to prevent the appearance of unwanted effects such as the development of tolerance and dependence. Because of the important role of NO in the development of morphine tolerance and dependence (43), we suggest determination of NO along with the endocannabinoid levels in the brain. As these effects can be modulated by the CB1 receptor (44), the use of cannabinoid receptor agonist and antagonist is necessary for further evaluation of PMSF effects on the development of morphine tolerance and dependence.
